# Research Ethics Recommendations for Whole-Genome Research: Consensus Statement

**DOI:** 10.1371/journal.pbio.0060073

**Published:** 2008-03-25

**Authors:** Timothy Caulfield, Amy L McGuire, Mildred Cho, Janet A Buchanan, Michael M Burgess, Ursula Danilczyk, Christina M Diaz, Kelly Fryer-Edwards, Shane K Green, Marc A Hodosh, Eric T Juengst, Jane Kaye, Laurence Kedes, Bartha Maria Knoppers, Trudo Lemmens, Eric M Meslin, Juli Murphy, Robert L Nussbaum, Margaret Otlowski, Daryl Pullman, Peter N Ray, Jeremy Sugarman, Michael Timmons

## Abstract

Interest in whole-genome research has grown substantially over the past few months. This article explores the challenging ethics issues associated with this work.

Advances in technology have made it possible to sequence a whole human genome [1,2]. National and international funding initiatives have stimulated whole-genome research activities [3,4], and media coverage of both the science [5,6] and the emerging commercial offerings [7,8] related to genome research has heightened public awareness and interest in personal genomics. As technology continues to advance, whole-genome research activities seem likely to intensify and expand, necessitating carefully considered consensus guidelines for ethical research practice.

To date, there has been only minimal consideration of the research ethics issues associated with this work [9–12]. We therefore convened an interdisciplinary consensus workshop to develop ethically rigorous and practical guidance for investigators and research ethics boards. What follows are recommendations on the four topics that were the focus of the workshop. These topics—consent, withdrawal from research, return of research results, and public data release—were selected because they were viewed as being among the most pressing research ethics issues and as representing areas where whole-genome analysis creates unique challenges. They are not, of course, the only policy issues that need to be considered; commercialization, patenting, benefit sharing, and the possibility of genetic discrimination are among other topics that warrant reflection. Regardless, the four topics covered in this paper are current issues worthy of immediate attention.

The paper starts with initial considerations, including general recommendations about governance and the characterization of the research activities related to the whole genome. It is important to note that while there was consensus on all recommendations, there was a good deal of debate about the degree to which they satisfy existing ethical and legal norms [13]. All participants believed that we need both empirical research and continued conceptual analysis (Box 1). These are early days in the field of whole-genome research. Research ethics guidance is needed immediately, but we should continue to explore the ethical, legal, and social implications of this rapidly evolving field (Box 2). Indeed, the door must remain open for further reflection on these and other social concerns.

**Box 1. Ethical Questions for the Future**

The following is a list of research ethics questions that warrant future investigation and analysis. It is not an exhaustive list of the research ethics questions associated with whole-genome research.

1. **Duty to Recontact:** Given the numerous disciplines and health care providers potentially involved in whole-genome research, further analysis of the legal and ethical obligations of each (particularly in the context of return of results) seems warranted. Who has what obligations? Who, if anyone, has the duty to return results? Is there a “chain of obligation” that runs through the various members of a research team?
2. **Right to Withdraw:** Because of the potential for the rapid dissemination of research results, the right to withdraw can quickly become impractical. This raises the issue of the degree to which there is an ethical requirement to structure the research and dissemination of results in a manner that will allow the right to withdraw to endure as long as possible. As such, this issue should be investigated with consideration of existing and evolving legal and ethical norms, emerging information technology tools that may facilitate withdrawal, and governance structures that can be implemented.
3. **Risk/Benefit Analysis:** There is a need for a comprehensive risk/benefit analysis of public data sharing. Studies that explore the impact on research of restricted access versus open access would be useful, and should include a consideration of costs and actual risk of harm.
4. **Governance Structures:** There is a need to systematically evaluate existing and emerging governance structures. This should include a consideration of the ways in which new information technologies can be utilized to facilitate, inter alia: continued communication with participants; the continued right to withdraw; and, when appropriate, community engagement.

**Box 2. Personal Genome Research: What Are the Possible Risks?**

**By Timothy Caulfield, Mildred Cho, and Amy McGuire**

The ability to sequence an individual's entire genome will allow for the production of an unprecedented amount of detailed genetic information, helping researchers to explore the relationship of genes and environment in the development of a wide variety of human diseases.

But imagine being a research participant in this exciting new field. Researchers would be seeking to produce a record of all your genetic information. As a result, all known genetic predispositions will be available and, depending on the data sharing policy, accessible to a wide range of researchers and, possibly, the public at large—this, at a time when we are still seeking to understand the social, clinical, and personal implications of genetic information.

These uncertainties can create unique ethical challenges. What do you tell potential participants during the consent process about risks when we still don't have a clear sense of their nature? Also, it is difficult to know, at the time of recruitment, exactly how the genetic information will be used, and by whom.

While most of the risks remain speculative, and we imagine that much of this research will be conducted by highly respected researchers at leading academic centers, one can imagine a number of controversial scenarios. Some of these may seem far-fetched, and we do not intend to be alarmist, but it is important to recognize that just one breach similar to those described below, or even the threat of such a breach, could hurt public trust and significantly hamper the ability to conduct genetic research.

Imagine you are watching the news and learn about a study linking race to IQ, which you find offensive. You later learn that they used your DNA for this study. You donated your DNA five years ago for use in a genetic association study of cancer and heart disease. At the time, you were told that other researchers might want to use your DNA for other types of research. You want to withdraw your consent, but it is too late. Your genetic information has been analyzed by many researchers and is now integrated in databanks throughout the world.

Imagine that you are sitting home, minding your own business, and the police show up at your door with a search warrant. They are looking for the suspect from a crime scene 2,000 miles away in Des Moines, Iowa. It turns out that some DNA that was left at the crime scene matched a sample from a publicly accessible scientific database that contained DNA from one of your brothers.

Imagine that at the time you first donated your sample for genetic research, it was explained as part of the consent process that no information about your genetics would be returned to you. Years later, you develop a heritable form of cancer and learn that the research team must have known you were genetically predisposed to the disease. To complicate matters further, you have a large number of siblings, none of whom want to know about their genetic predispositions. This information is now available to all.

Imagine you just opened your own electronics recycling business and are trying to find private health insurance. Your friend recommends that you contact a specific company. As part of the enrollment process, the company finds out that you participated in a genetic study and that your genetic information has been released into a publicly accessible database. By accessing the database, the company finds out that you are at increased risk of early-onset Alzheimer disease and are highly susceptible to cancer from the chemicals encountered in your recycling process. They also find that you are at greatly increased risk of colon cancer, which alerts you to now take early screening and preventive measures.

This is, no doubt, an exciting time for genetic research. And it cannot move forward without research participants. As such, it is important to note that the risks associated with this kind of research may be limited and controversial events rare. But history has told us that they do occur and can have a devastating impact on public trust and the research environment. It is therefore critical that as this research moves forward, there are guidelines in place, such as those outlined by this paper, to promote ethical research conduct and to help avoid, as much as possible, scenarios like those described above.

## Initial Considerations, Governance, and Oversight

Whole-genome research can be utilized in a wide variety of contexts, including as a mode to advance sequencing technology, to develop a research resource platform, and to do disease-specific investigations. Regardless of the purpose of the activity, whole-genome research may implicate issues and ethics norms associated with population health (particularly when used in conjunction with biobanks and cohort studies), the provision of health care, and research. Recognizing that the characterization and resolution of ethical challenges will vary depending on which domain is used as the lens of analysis, we explicitly chose to consider the following issues from the perspective of research ethics. This lens was chosen because most of the current work seems to be research activities and, for the purpose of governance and regulation, should be characterized as such. In addition, given the preliminary nature of the technology, it was felt that it was prudent to frame whole-genome sequencing as a biomedical research enterprise, and more specifically as research involving human subjects.

As with other emerging areas of research (e.g., biobanking initiatives) [14,15], whole-genome sequencing tests traditional clinical trials ethics paradigms, particularly in the areas discussed below. For example, the vast amount of data produced, the uncertain future research uses of the data, the implications of the data for family members, and the technological ability (and expectations) to publicly release the data are realities that challenge existing research ethics norms. While these issues have emerged in other contexts [16,17], they seem particularly acute in the context of whole-genome research. For example, this research will produce an amount of genetic information about an individual well beyond what is currently done in most genetic research protocols. Also, given the recent rapid advances and intense interest in whole-genome research, it seems worthwhile to consider how existing research ethics norms apply.

**Figure 1:**
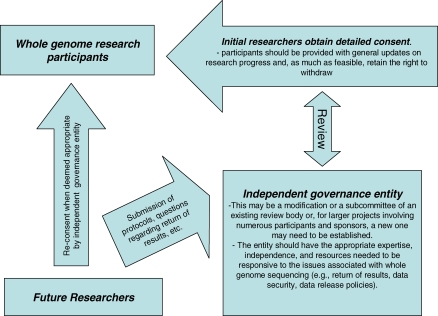
Example Governance Structure The following governance structure is similar to other policies [22]. It is only an example and should be modified to meet the needs and scope of each research initiative. The mandate and scope of the entity's responsibilities, obligations, and powers should be clear and should be reconciled with roles and requirements of existing ethics review committees. The membership of the entity, their criteria and process for making decisions, and the review and appeal system should be publicly available in order to ensure transparency and accountability.

This area of research invites a move beyond reliance on the usual informed consent strategies [18], to the utilization of robust governance and oversight mechanisms [19]. Ideally, the governance scheme should have a mandate to ensure research integrity and the protection of the interests of all stakeholders, including the public, participants, family members, communities, and the research community at large. Governance structures should reflect the nature and scope of the particular whole-genome research protocol (Figure 1). A project involving a single research participant would have a more modest governance framework than an initiative engaging a large number of individuals or a particular population or community. Also, in designing governance structures, investigators should draw on existing ethics resources (e.g., local committees and experts). Attention has to be paid to concerns that research ethics review structures may not always be sufficiently independent and that improvements to the system are needed to promote their accountability [20]. Regardless, as will be discussed, appropriate governance schemes seem essential to the ethical conduct of whole-genome investigations—in part because the unique challenges associated with the research make it impractical to satisfy the norms, tools, and processes usually utilized to respect autonomy (e.g., specific informed consent). Also, the ethical issues often move beyond autonomy challenges alone [21].

## Consent

Whole-genome research involves the collection of a biological sample, the sequencing of the genome, various levels of data analysis, and, possibly, the use of the sample and/or data for a wide variety of future research projects that are likely unknown at the time that the sample is taken. In addition, the data may be released into scientific databases that are publicly accessible in order to facilitate research (to be discussed below). These activities create tremendous consent challenges.

First, the implications and potential social risks associated with the sequencing of an entire genome remain unknown. While most of the data will have limited immediate clinical significance, the massive volume of the data triggers challenges to protecting privacy and respecting autonomy. In addition, as will be discussed below, whole-genome research creates complex ethics challenges associated with the repercussions of data release and return of results, all of which must be covered in the initial informed consent process. The involvement of commercial entities and the potential commercialization of research results complicate the process even more. Given the uncertainty and complexity of the activity, ensuring fully informed consent will be difficult.

Second, the fact that there will likely be many unspecified future uses for the data raises the question of whether reconsent is required. Given that numerous investigators may be using the data over an extended period, reconsenting all individuals who participate in whole-genome research for all uses of the data seems impractical—a challenge that has been encountered in other areas of research, such as biobanking [13,22].

Finally, when individuals provide consent for whole-genome scanning of their own biological material, this also has implications for family members and community members with whom they share some genetic information. Traditional individually based informed consent procedures do not deal well with this aspect of genetic research.

Informed consent is a foundational principle in research ethics [23,24]. It is meant to respect individual autonomy. While it is recognized that consent remains essential, in the context of whole-genome research it is not likely that it can be fully satisfied nor do all the work. As such, the consent process must be supplemented with a governance structure that can respond to the particular ethics issues associated with whole-genome research.

### Recommendation 1.

Prior to participation in a whole-genome project, participants should be asked to provide consent for future use that includes as much detail as possible, including information about the sampling and sequencing process, associated commercialization activities, possible risks, and the nature of likely future research initiatives. The consent process should also include information about data security and the governance structure and, in particular, the mechanism for considering future research protocols (Figure 1). When deemed appropriate by the governance scheme, reconsent for specific research projects may be required (e.g., when the proposal deviates significantly from what was stated in the initial consent).

## Withdrawal from Research

The right of research participants to withdraw consent at any time, for any reason and without repercussions is a central component of existing research ethics statements [23–25]. In general, this right extends to research on identifiable health information and tissue samples [26]. In the context of whole-genome research, this right endures and must be respected. Indeed, any minimization of the right to withdraw could pose a threat to public trust. However, because whole-genome research results in the production of data that, when used for research, are likely to be disseminated rapidly and widely (depending on the relevant data release policy), there will inevitably be profound practical limitations to the right to withdraw. Indeed, once whole-genome research data are released, it will be very difficult, if not impossible, to retrieve or destroy the data in response to a withdrawal request.

So, while research participants retain the right to withdraw their consent whenever possible (e.g., they could request the destruction of the relevant tissue sample or the severance of ongoing linkages to personal information), it will often be impossible to destroy data that have already been released. As part of the ethics review process, there should be a careful consideration of how far along the research process withdrawal can (and should) be possible. This should also be done in order to inform the consent process and to ascertain the proper balance between research goals and the right to withdraw.

### Recommendation 2.

The right to withdraw consent, including the destruction of tissue samples and written information, must, so far as possible, be respected and be part of the whole-genome research ethics process. In addition, the fact that this right may be severely limited once data are disseminated must be clearly communicated as part of the initial informed consent process.

### Recommendation 3.

The design of personal genome projects and ethics review should explicitly consider how the ability to withdraw from subsequent use is enhanced or diminished by how data and samples are collected, stored, and disseminated. The appropriate balance will need to be considered for each project on a case-by-case basis.

## Return of Results

Even if the purpose of some whole-genome research is not to provide results about individual participants, the work may generate such results, ranging on a continuum from clinically significant information to information relevant to ancestry and genealogy to information that is merely of recreational interest.

On this continuum, there may be situations in which the research generates genetic information that the researcher has a moral (but not necessarily legal) obligation to offer to individual participants [27,28]. Such research may also generate results that could be detrimental or that a research participant has specifically asked not to be returned. Due to the unknown clinical value of much of the genomic data, the amount of information that a researcher might have a duty to disclose will likely be small at the current time. However, it is expected to grow with increased knowledge about the clinical significance of the genome. In addition, some associations will be found by other research teams, warranting review and tough decisions about whether to invest time in confirming results for one's own study population and informing subjects about those secondary findings [29].

The duty to offer results is, in part, related to the relationship between researchers and participants. It may also be based on expectations founded on institutional or cultural assumptions. Because results may be generated by researchers who did not collect the samples and who may not know the identity of the research participants, there are interesting questions about who may have a duty to offer results. Do researchers who provide samples or data to other researchers for secondary analyses retain responsibility to support return of appropriate results?

### Recommendation 4.

Personal genome research projects should have an established process, approved by a research ethics review entity, for evaluating whether findings (incidental or otherwise) meet criteria for offering to individual participants (Figure 1). This process should be highlighted in the initial consent and should acknowledge the participants' right not to know certain results.

### Recommendation 5.

The process of identifying and disclosing research results should involve professionals with the appropriate expertise required to provide the participant with sufficient interpretive information. In general, the results offered should be scientifically valid, confirmed, and should have significant implications for the subject's health and well-being. Plans to return other forms of data—such as significant non-health-related data—should be built into the study design and governance structure.

## Public Data Release

International policies call for the rapid public release of all sequence data [30–32]. The benefit of public data access is that it provides significant scientific utility by enabling immediate international research use of the data. However, policies that advocate unrestricted data sharing have been challenged because of the privacy risks associated with public access to genomic information [33,34]. Whole-genome research increases these privacy concerns, particularly the uncertainties surrounding the implications of the data. As a result, a cautious approach seems warranted. Investigators and research ethics boards need to carefully consider whether public data release is warranted.

As noted above, once data are released into a publicly accessible database, it becomes impossible to withdraw the data from the public domain. The finality of public data sharing needs to be clearly articulated in the informed consent process. A balanced approach should be adopted when explaining the risks and benefits of data sharing. In the consent and review process, neither the benefits of research nor the potential privacy risks should be minimized or exaggerated. In this regard, several potential privacy issues should be discussed: the data security mechanisms put in place; the primary concern of identifiability; the practical limits on an individual's right to control access to their personal information; and the secondary concern about genetic profiling and an individual's right to control their “ascribed social identity” [35]. Given the complexity of these privacy challenges, it is essential that the relevant ethics review entity have appropriate ethics expertise.

More research and policy analysis on the issues associated with data release is clearly needed, including an analysis of the actual harms and benefits resulting from publicly accessible data; the implications for family members and relevant communities; the appropriate balance between public access and individual privacy interests; and considerations regarding compensation for research-related injury resulting from participation in personal genome research. While the likelihood of injury is small, compensating individuals for harm or loss associated with research participation would promote reciprocity for participation in research with no direct benefit to the individual.

It may be more appropriate to release genome data into databases with restricted access. Restricted access databases typically require some authentication so that only bona fide researchers can access the information. Most restricted access databases provide some phenotypic information linked to the genotypic data, increasing the scientific utility of the data. Restricted access provides enhanced privacy protection, but the level of restriction and stringency of oversight vary significantly depending on the database (e.g., the degree to which the database contains sensitive or identifying information). Regardless, appropriate security, oversight, and access policies are essential for the protection of human subjects.

### Recommendation 6.

Data release policies must be designed to appropriately balance the benefits and requirements of access against the privacy interests of research participants. The rationale for the proposed data release policy needs to be clearly explained, justified as necessary for the goals of research, and deemed acceptable by the relevant ethics review entity.

### Recommendation 7.

The implications of data release must be adequately disclosed to the potential participants in the consent process. This disclosure should include a discussion of the likely finality of the release process and the implications that this may have on privacy and the future right to withdraw.

### Recommendation 8.

As part of the consent and ethics review process, the issues associated with family members and relevant groups and populations should be considered (this may, for example, involve encouraging/requiring discussions with family members).
